# Effects of match contextual factors on internal and external load in elite Brazilian professional soccer players through the season

**DOI:** 10.1038/s41598-022-25903-x

**Published:** 2022-12-09

**Authors:** Rodrigo Aquino, Rodrigo Guimarães, Geraldo Oliveira Carvalho Junior, Filipe Manuel Clemente, Tomas García-Calvo, Juan José Pulido, Hadi Nobari, Gibson Moreira Praça

**Affiliations:** 1 LabSport, Post-Graduate Program in Physical Education, Centre of Physical Education and Sports, Federal University of Espírito Santo, Vitória, Brazil; 2 Department of Physiology, América Football Club, Belo Horizonte, Brazil; 3 Programa de Doctorado en Ciencias del Deporte, Facultad de Ciencias del Deporte, Universidad de Extremadura, 10071 Cáceres, Spain; 4 Escola Superior Desporto e Lazer, Instituto Politécnico de Viana do Castelo, Rua Escola Industrial e Comercial de Nun’Álvares, 4900-347 Viana do Castelo, Portugal; 5 Research Center in Sports Performance, Recreation, Innovation and Technology (SPRINT), 4960-320 Melgaço, Portugal; 6 Instituto de Telecomunicações, Delegação da Covilhã, 1049-001 Lisboa, Portugal; 7 Faculty of Sport Sciences, University of Extremadura, Avenida de la Universidad, 10003 Cáceres, Spain; 8 Faculty of Education and Psychology, University of Extremadura, Avenida de Elvas, 06006 Badajoz, Spain; 9 Department of Exercise Physiology, Faculty of Educational Science and Psychology, University of Mohaghegh, Ardabil, 56199-11367 Iran; 10 Department of Motor Performance, Faculty of Physical Education and Mountain Sports, Transilvania University of Braşov, 500068 Braşov, Romania; 11 Universidade Federal de Minas Gerais, Belo Horizonte, Brazil

**Keywords:** Physiology, Medical research, Engineering

## Abstract

This study aimed to investigate the effects of contextual match factors (quality of opposition, match outcome, change of head coach or playing style) on internal and external load in elite Brazilian professional soccer players, considering the total and effective playing time. Twenty-two professional male outfield soccer players participated in this study (age 28.4 ± 4.9 years; height 1.78 ± 0.1 cm; body mass 72.9 ± 7.1 kg). The internal (rating of perceived exertion-based load [sRPE]) and external load (distance and accelerometry-based measures) were recorded during 38 matches, over the 2021 season of the Brazilian National 1st Division League using a global position system (10 Hz) integrated with an accelerometer (200 Hz). The main results were: (i) matches played against weak opponents presented greater values of sprinting distances compared to matches against intermediate and strong opponents; (ii) players covered greater high-intensity running distances when drawing than winning the matches; (iii) matches with assistant coaches presented higher mean speed relative to effective playing time (MSEPT) compared to coach 1 and coach 2 conditions. In addition, players covered greater MSEPT and high-acceleration in matches with coach 2 vs. coach 3; (iv) finally, small positive correlations were observed between positional attack sequences and MSTPT, total distance covered, and acceleration. Coaches and practitioners should consider these results when interpreting external load variables during elite Brazilian soccer matches.

## Introduction

The soccer match is characterized by an intermittent effort, in which a predominance of low-to-moderate physical activities is interspaced by maximum-to-near maximum efforts^1^. Male professional soccer players typically cover 9–14 km during a match^2^, from those 600 to 1100 m can be covered at high-intensity running (running > 19.8 km/h)^3^ and 250–300 m can be covered at sprinting (running faster than 25 km/h)^4^. Moreover, it is also known that male professional soccer players may perform 1400 activities and 700 directional changes, attain more than 600 accelerations and 600 decelerations, and perform up to 40 very high-intensity efforts (running > 21 km/h)^2^. The relationship between work and rest in a match is typically about 1:12 (ratio) considering the rest and high-intensity efforts, although in intense periods may drop to 1:2^2^.

The information about the psychophysiological, locomotor, and mechanical impact of the match on male professional soccer players can be critical for managing the recovery strategies^5^ and, importantly, adjusting the training interventions to their requirements^6^. Thus, monitoring players’ psychophysiological, locomotor, and mechanical demands have become a prevalent practice in male professional soccer^7–9^. A systematic review revealed that the use of objective instruments such as the Global Positioning System (GPS) or subjective questionnaires as the rate of perceived exertion (RPE) are frequently employed by coaches and practitioners^10^. As an example, a study conducted on fifty-two strength and conditioning coaches working in professional soccer revealed that GPS devices are used in 94% of the cases^11^. In addition, previous studies reported that the differential rating perceived exertion (dRPE) can be a valid method for assessing internal training and match load in soccer^12,13^.

Typical psychophysiological, locomotor, and mechanical demands attained in a match can be constrained by contextual factors (also known as situational factors)^14,15^, naturally emphasizing the importance of monitoring systematically. Among those contextual factors, we can consider the match location (playing at home vs. away), competitive level, quality of opponents team, team's formation, playing position, match status, final score, or tactical behaviors^16–19^. As an example, a study conducted on Brazilian male professional soccer players revealed that playing at home or playing against weaker opponents conducted to greater locomotor demands^16^. Similar results were found in Portuguese male professional players^20^.

Usually, contextual factors such as match outcome are the most prevalent reported in the scientific community^16–18^. However, other contextual factors such as the presence or not of assistant coaches or the effect of effective playing time^21^ can also interact with the final demands experienced by players. Despite that, few research has been centered on such a contextual factor. Moreover, evidence is needed to identify the weight of these factors for the final experienced psychophysiological, locomotor, and mechanical demands of players. This could help practitioners and coaches to better estimate the impact on the players and guide them for training strategies based on the contexts that will face.

Therefore, this study aimed to investigate the effects of contextual match factors (quality of opposition, outcome, change of head coach or playing style) on internal and external load in elite Brazilian professional soccer players, considering the total and effective playing time. We hypothesize that greater locomotor and mechanical demands will occur in home matches and playing against weaker opponents.

## Methods

### Design

The players' running performance was quantified during the matches over the 2021 season of the Brazilian National 1st Division League. This League was disputed by 20 soccer clubs, in-home and away matches, totalizing 38 matches. The reference team observed was positioned in the top-10 position in the general ranking, obtaining a spot in CONMEBOL Libertadores 2022. This study investigated the effects of quality of opposition (three clusters: strong [1st to 4th position] vs. intermediate [5th to 15th position] vs. weak [16th to 20th position]), match outcome (win vs. draw vs. loss), change of head coach (assistant coach vs. coach 1 vs. coach 2 vs. coach 3), and playing style (counterattack vs. positional attack) on a myriad of internal (RPE) and external load (distance- and accelerometry-based measures) during the official matches. The total playing time presented a mean of 98 min (std. deviation = 3). The effective playing time presented a mean of 49 min (std. deviation = 6). The matches were played between 11:00 a.m. and 9:30 p.m. times.

### Participants

Twenty-two professional male outfield soccer players participated in this study (age 28.4 ± 4.9 years; height 1.78 ± 0.1 cm; body mass 72.9 ± 7.1 kg; central defenders = 4; external defenders = 5; midfielders = 7; forwards = 6; range of individual match observations: 1–29). Inclusion required participation in ≥ 90 min of play. The study was approved by the local Human Research Ethics Committee (Centre of Physical Education and Sports, Federal University of Espírito Santo: 10954/2021).

### Dependent variables

External load: The distance- and accelerometry-based measures were recorded during the matches using a wearable 10-Hz GPS integrated with a 200-Hz Tri-Axial accelerometer, gyroscope, and magnetometer (Polar Electro, Kempele, Finland). The validity and accuracy of the devices were previously reported in the literature^22^. The devices were fitted to the upper back of each player using adjustable harnesses and were activated 15 min before the data collection, in accordance with the manufacturer's instructions to optimize the acquisition of satellite signals. Throughout the season, the players used the same device to avoid inter-unit errors^23^. The following metrics were obtained: (i) total distance covered (TD, m); (ii) total distance covered under moderate-intensity running (MIR, 14.4–19.8 km h^−1^), (iii) total distance covered under high-intensity running (HIR, 19.8–25.2 km h^−1^), (iv) total distance covered under sprinting running (SPR, > 25.2 km h^−1^, m); (v) maximal running speed (MRS; km h^−1^); (vi) mean speed relative to total playing time (MSTPT; m min^−1^); (vii) mean speed relative to effective playing time (MSEPT; m min^−1^); (viii) total distance covered under high-acceleration (Acc, ≥ 3 m s^−2^, m); (ix) total distance covered under high-deceleration (Dec, ≤  − 3 m s^−2^, m). The speed and accelerometry thresholds used are similar to those reported in a previous systematic review^24^.

Internal load: The Borgs' scale (CR10) was previously presented to the team for familiarization on a daily basis for one month. Approximately thirty minutes after the end of the matches, players were asked to answer "How was your workout?". Internal load (rating of perceived exertion-based load [sRPE]), reported as arbitrary units (AU), was calculated by multiplying the adapted version of Borg's CR10 score^25^ by the total match duration^26^. The sRPE is considered a valid indicator of internal load in soccer^27^.

### Independent variables

Four contextual factors were included in this study: (i) quality of opposition—determined according to k-means cluster analysis based on the final ranking. The grouping is performed by minimizing the sum of squares of distances between data and the corresponding cluster centroid, which is the arithmetic mean for each dimension separately over the ranking differences in the cluster^28^. The results identified 3 clusters: “best ranking,” featuring strong opponents (1st to 4th position; n = 44 individual observations); “medium ranking”, including the intermediate opponents (5th to 16th position; n = 126 individual observations), and “worst ranking” representing the weak opponents (16th–20th position; n = 42 individual observations); (ii) match outcome—win (n = 74 individual observations), draw (n = 83 individual observations), and loss (n = 55 individual observations); (iii) change of head coach—including the assistant coach (n = 16 individual observations), coach 1 (n = 10 individual observations), coach 2 (n = 118 individual observations), and coach 3 (n = 68 individual observations). At least two matches as head coach were considered as inclusion criteria; (iv) playing style—counterattack (i.e., number of offensive sequences that includes a quick transition/direct play, trying to take advantage of the opponent being out of position; n = 137 sequences) vs. positional attack (i.e., number of offensive sequences than any open play attack/indirect play that is not considered a counterattack; n = 1165 sequences). The analysis of playing style was obtained using the Wyscout reports (Wyscout, Chiavari, Italy), with the reliability confirmed in a previous study^29^.

### Statistical analysis

Kolmogorov–Smirnov test was used to check the data distribution normality and no violations were detected. Data are expressed as mean, standard deviation, minimum and maximum. To account for the non-independence of data sampled from the same individuals across multiple matches separate linear mixed models were performed to compare (fixed effects) quality of opposition (top four vs. middle twelve vs. bottom four), match outcome (win vs. draws vs. losses), and change of head coach (assistant coach vs. coach 1 vs. coach 2 vs. coach 3) with "player ID" included as a random effect. Furthermore, multiple comparisons were adjusted using the Bonferroni method. The t-statistics from the mixed models were converted to effect size correlations^30^. In addition, the magnitude of the correlation between playing style and internal/external load was analyzed using the Pearson product test (CI 95%). The effect size and correlation coefficients (r) were classified as follows: trivial (r < 0.1), small (r = 0.1–0.3), moderate (r = 0.3–0.5), large (r = 0.5–0.7), very large (r = 0.7–0.9), and almost perfect (r > 0.9)^31^. A significance level of p < 0.05 was adopted. Data were analyzed using the software SPSS Statistics for Windows, version 22.0.

### Declarations

The training coaches of the club, after obtaining permission from the relevant authorities and the head coach of the club, conducted this research. This study received the approval of the research ethics committee from the Federal University of Espírito Santo (10954/2021). All players were informed of the purpose of the study before completing the informed consent and informed consent has been obtained from all study participants. All stages of this study were carried out based on the ethical principles in the Helsinki Declaration.

## Results

### Quality of opposition

Matches played against weak opponents presented reduce values of MSEPT compared to matches played against intermediate opponents (t = 2.410; *p* = 0.02; ES = 0.21, small). On the other hand, matches played against weak opponents presented greater values of SPR compared to matches against intermediate (t = 2.617; *p* = 0.02; ES = 0.60, large) and strong opponents (t = 3.101; *p* = 0.007; ES = 0.62, large). Non-significant differences were detected for any other performance variable (*p* > 0.05; Table 1).

**Table 1:** Effects of quality of opposition on internal and external load during official matches in elite Brazilian soccer players.

	sRPE (AU)			MSTPT (m min^−1^)			MSEPT (m min^−1^)			MRS (km h^−1^)			TD (m)			MIR (m)			HIR (m)			SPR (m)			Acc (m s^2^)			Dec (m s^2^)		
	W	I	S	W	I	S	W	I	S	W	I	S	W	I	S	W	I	S	W	I	S	W	I	S	W	I	S	W	I	S
Mean	372.3	365.2	377.0	101.8	100.6	100.9	197.8*	207.3	202.0	35.0	33.9	33.7	10,080.4	9853.9	10,020.1	1284.2	1263.0	1194.0	460.0	434.4	401.9	177.9**	144.4	122.1	28.7	26.7	28.3	28.7	27.8	30.1
Std. Deviation	59.8	60.3	52.4	10.1	10.5	10.0	28.9	30.7	27.1	5.25	3.6	4.2	958.2	1003.1	963.1	404.5	483.1	421.4	172.9	200.1	160.0	93.4	101.8	58.3	9.7	8.2	9.0	7.8	11.2	9.6
Minimum	184.0	178.0	212.0	80.4	72.4	84.5	135.3	145.7	151.2	29.5	26.3	26.8	8523.0	7242.0	8401.0	623.0	442.0	504.0	187.0	89.0	77.0	39.0	8.0	12.0	14.0	7.0	9.0	12.0	6.0	8.0
Maximum	481.0	473.0	461.0	124.0	129.1	124.8	265.6	314.6	271.7	53.9	44.1	49.0	12,291.0	12,460.0	12,252.0	2158.0	2835.0	2545.0	912.0	1135.0	721.0	427.0	537.0	347.0	50.0	49.0	57.0	47.0	55.0	57.0

*W* Weak, *I* Intermediatie, *S* Strong, *AU* arbitraty units, *sRPE* session of rating of perceived exertion, *MSTPT* mean speed relative of total playing time, *MSPT* mean speed relative of effective playing time, *MRS* maximal running speed, *TD* total distance covered, *MIR* moderate intensity running (14.4–19.8 km h^−1^), *HIR* high intensity running (19.8–25.2 km h^−1^), *SPR* sprinting (> 25.2 km h^−1^), *Acc* high acceleration (> 3 m s^2^), *Dec* high deceleration (< − 3 m s^2^). *Weak < intermediate. **Weak > intermediate and strong.

### Match outcome

Players covered lower MIR distances when win compared to loss (t = 2.669; *p* = 0.009; ES = 0.23, small) and draw matches (t = 4.250; *p* < 0.001; ES = 0.58, large). In addition, players covered greater HIR distances when draw than win the matches (t = 4.054; *p* < 0.001; ES = 0.64, large). Non-significant differences were identified for any other variable (*p* > 0.05; Table 2).

**Table 2:** Effects of match outcome on internal and external load during official matches in elite Brazilian soccer players.

		sRPE (AU)						MSTPT (m min^−1^)						MSEPT (m min^−1^)						MRS (km h^−1^)						TD (m)						MIR (m)						HIR (m)						SPR (m)						Acc (m s^2^)						Dec (m s^2^)					
		L		D		W		L		D		W		L		D		W		L		D		W		L		D		W		L		D		W		L		D		W		L		D		W		L		D		W		L		D		W	
Mean		361.4		367.8		376.3		101.4		100.8		100.6		204.3		202.5		206.3		33.3		34.1		34.6		9927.9		9959.8		9907.5		1251.1		1336.3		1160.7*		422.0		477.8		390.2**		136.8		159.0		139.4		28.2		26.9		27.6		28.2		30.2		26.7	
Std. Deviation		57.4		58.4		59.6		10.4		11.2		9.1		30.0		36.2		20.2		3.4		4.5		4.1		1012.2		1038.3		917.9		425.5		519.2		381.6		175.1		220.4		141.3		77.9		112.0		81.9		8.8		7.9		9.5		8.9		10.4		10.9	
Minimum		202.0		193.0		178.0		84.5		72.4		80.0		151.2		135.3		163.3		26.8		26.3		29.3		8401.0		7242.0		8162.0		504.0		546.0		442.0		77.0		166.0		166.0		12.0		8.0		19.0		9.0		10.0		7.0		8.0		8.0		6.0	
Maximum		475.0		481.0		461.0		124.8		129.1		121.5		271.7		314.6		265.6		44.5		53.9		50.0		12,291.0		12,460.0		11,987.0		2545.0		2835.0		1986.0		837.000		1135.0		685.0		427.0		537.0		420.0		57.0		47.0		49.0		57.0		55.0		52.0	

*L* Loss, *D* Draw, *W* Win, *AU* arbitraty units, *sRPE* session of rating of perceived exertion, *MSTPT* mean speed relative of total playing time, *MSPT* mean speed relative of effective playing time, *MRS* maximal running speed, *TD* total distance covered, *MIR* moderate intensity running (14.4–19.8 km h^−1^), *HIR* high intensity running (19.8–25.2 km h^−1^), *SPR* sprinting (> 25.2 km h^−1^), *Acc* high acceleration (> 3 m s^2^), *Dec* high deceleration (< − 3 m s^2^). *Win < draw and loss. **Draw > win.

### Change of head coach

Coach 1 condition presented reduce values of MSEPT than Assistant Coach (t = 4.712; *p* < 0.001; ES = 0.38, moderate) and Coach 2 conditions (t = 3.867; *p* < 0.001; ES = 0.29, small). Assistant Coach condition also presented higher MSEPT compared to Coach 2 (t = 2.257; *p* = 0.02; ES = 0.17, small) and Coach 3 conditions (t = 4.568; *p* < 0.001; ES = 0.37, moderate). Coach 2 condition presented greater values of MSEPT (t = 4.241; *p* < 0.001; ES = 0.31, moderate) and Acc (t = 4.006; p = 0.006; ES = 0.84, very large) than Coach 3 condition. Non-significant differences were identified for other running performance variable (*p* > 0.05; Table 3).

**Table 3:** Effects of change of head coach on internal and external load during official matches in elite Brazilian soccer players.

Variables	Staffs	Mean (SD)	Minimum	Maximum
sRPE (AU)	Coach 1	367.6 (54.5)	301.0	445.0
	Assistant coach	360.4 (55.5)	258.0	450.0
	Coach 2	365.9 (62.8)	178.0	481.0
	Coach 3	376.7 (52.3)	238.0	461.0
MSTPT (m min^−1^)	Coach 1	104.4 (10.9)	90.4	124.8
	Assistant coach	101.2 (11.6)	84.5	119.0
	Coach 2	101.0 (9.9)	80.4	127.8
	Coach 3	100.2 (10.5)	72.4	129.1
MSEPT (m min^−1^)	Coach 1	182.4 (21.4)	151.2	216.5
	Assistant coach	225.2 (26.2)	185.3	268.7
	Coach 2	207.1 (32.6)	135.3	314.6
	Coach 3	197.8 (21.8)	155.6	255.6
MRS (km h^−1^)	Coach 1	33.0 (4.4)	27.9	41.2
	Assistant coach	33.6 (4.3)	26.8	42.1
	Coach 2	34.2 (4.2)	26.3	53.9
	Coach 3	34.2 (3.9)	26.9	50.0
TD (m)	Coach 1	10,079.2 (1045.7)	8771.0	11,984.0
	Assistant coach	10,029.9 (1171.9)	8573.0	12,252.0
	Coach 2	9921.8 (949.2)	8188.0	12,460.0
	Coach 3	9909.1 (1015.4)	7242.0	12,261.0
MIR (m)	Coach 1	1410.2 (510.4)	785.0	2545.0
	Assistant coach	1246.8 (528.1)	504.0	2243.0
	Coach 2	1222.6 (486.6)	442.0	2835.0
	Coach 3	1283.8 (367.8)	678.0	1995.0
HIR (m)	Coach 1	402.6 (199.4)	89.0	721.0
	Assistant coach	411.8 (239.1)	77.0	919.0
	Coach 2	422.1 (199.4)	166.0	1135.0
	Coach 3	460.6 (147.4)	205.0	852.0
SPR (m)	Coach 1	87.1 (45.8)	13.0	179.0
	Assistant coach	115.3 (69.1)	12.0	247.0
	Coach 2	142.9 (89.5)	8.0	537.0
	Coach 3	168.6 (106.0)	42.0	503.0
Acc (m)	Coach 1	26.7 (6.4)	15.0	39.0
	Assistant coach	27.8 (9.0)	9.0	41.0
	Coach 2	28.9 (8.5)	11.0	57.0
	Coach 3	24.9 (8.8)	7.0	44.0
Dec (m)	Coach 1	28.2 (13.5)	10.0	57.0
	Assistant coach	25.0 (10.7)	8.0	43.0
	Coach 2	28.8 (9.5)	6.0	55.0
	Coach 3	28.7 (11.1)	6.0	53.0

*AU* arbitraty units, *sRPE* session of rating of perceived exertion, *MSTPT* mean speed relative of total playing time, *MSPT* mean speed relative of effective playing time, *MRS* maximal running speed, *TD* total distance covered, *MIR* moderate intensity running (14.4–19.8 km h^−1^), *HIR* high intensity running (19.8–25.2 km h^−1^), *SPR* sprinting (> 25.2 km h^−1^), *Acc* high acceleration (> 3 m s^2^), *Dec* high deceleration (< − 3 m s^2^). *Coach 1 < Assistant Coach and Coach 2. **Assistant Coach > Coach 2 and 3. ***Coach 2 > Coach 3.

### Playing style

Non-significant correlations were verified between counterattack sequences and internal and external load variables (r = − 0.12–0.11 [95% CI − 0.24–0.22]; *p* = 0.07–0.99). Small correlations were identified between positional attack sequences and MSTPT (r = 0.14 [95% CI 0.004–0.27]; *p* = 0.04), TD (r = 0.14 [95% CI 0.006–0.27]; *p* = 0.04), and Acc (r = 0.16 [95% CI 0.04–0.28]; *p* = 0.02).

## Discussion

This study investigated the effects of contextual match factors on internal and external load in elite Brazilian professional soccer players, considering the total and effective playing time. The main results were: (i) matches played against weak opponents presented greater values of sprinting distances (SPR) compared to matches against intermediate and strong opponents; (ii) players covered greater HIR distances when drawing than winning the matches; (iii) matches with assistant coaches presented higher mean speed relative to effective playing time (MSEPT) compared to coach 1 and coach 2 conditions. In addition, players covered greater MSEPT and high-acceleration (Acc) in matches with coach 2 vs. coach 3; (iv) finally, small positive correlations were observed between positional attack sequences and MSTPT, total distance covered (TD), and Acc.

Playing against weak opponents increased players' sprints in the matches. Previous studies in the literature revealed that playing against weaker teams increases ball possession^32,33^, increases defensive performance^34^, and increases the team's offensive territorial domain^35^. These results indicate that playing under this condition increases teams' performance and creates the opportunity to occupy larger areas in the field. Consequently, players have to sprint more to cover these larger areas, which explains the current results. However, we acknowledge that our results are contrary to a previous study that reported higher total distance covered when playing against stronger teams and no differences in the sprint performance^36^. The characteristics of the sample might explain the differences in the result. While Rampinini et al.^36^ analyzed a top-level team, the athletes from the current sample belong to a mid-table team. In this context, we speculate that players engaged in a similar number of matches against weak or strong opponents in the present study. In the previously cited study, however, players were probably involved in a few games against strong opponents (compared to their actual performance). Consequently, the few matches against strong opponents could be raised extra motivation and increase physical performance, which explains the differences in the results. Finally, no differences in most of the analyzed variables were reported, which indicates that even if the quality of opposition affects performance, it seems not to be a significant factor in explaining match-to-match performance variations in the Brazilian national league.

Higher values of high-intensity runs and sprints were reported in won matches. Previous results showed that winning games presented higher distances covered by the players, who also performed more accelerations than in losing games^20^. In another study, players from winning teams spent more time in higher intensities (which equals the current threshold for sprinting)^37^, similar to the current results. Also, forwards and central defenders covered higher distances above 24 km/h when they won the matches^38^. Even if match status was considered in the current study instead of match outcome, a recent study found a similar trend of higher physical responses when winning than losing statuses were compared^39^. In summary, achieving high physical responses, mainly in high-intensity actions, is a well-established feature of winning teams. Specifically, the possibility of winning sprinting duels and covering larger areas, achieved by players who present higher physical responses, explains the current results. Finally, this trend seems to be influenced by playing position^40,41^. Therefore, we recommend future studies with larger samples, making it possible to split the data by players' playing positions.

Changing the head coach is a common feature in elite soccer. There were 28 and 21 changes in the last two years (2020, and 2021, respectively) in the 1st Division of the Brazilian national championship. Independently of the reason (poor performance or the coach's own decision to leave the club), there is a change in the training routine and match strategy that can affect players' and teams' performances. Most of the dependent variables showed no differences in the current study when players' performances were compared across the conditions. At this point, the literature showed nil long-term effects of changing the head coach^42–44^. However, short-term performance increases might be observed^44,45^, which could also be explained by players' expected performance variation^46^. Concerning physical performance, another study showed that only training performance indicators were affected by the change of the head coach, with no differences in match-related indicators^47^. Therefore, it is possible to assume that the expected impact of changing the head coach is not usually achieved in the long term. Players' difficulty in dealing with changes in the training process, the need to update game principles, and the few available training time in midseason might be the most reasonable reasons for such a nil effect.

In the current study, we included the data related to those matches in which the assistant coach was in charge. At this point, we observed a higher mean speed relative to effective playing time when the assistant coach was in charge. Specifically, the assistant coach usually is a professional who is kept in the club when the head coach is dismissed or decides to leave the club. Therefore, this professional can keep regular training routines and match strategy, which constitutes a positive constraint to players' performance. As we have previously shown that winning^37^ and playing against weak opponents^32,33^ increases physical performance, it is arguable that replacing the head coach with a professional who is aware of the team routines is likely to increase the performance in comparison to selecting a completely different coach. Future studies at this point are recommended to enlarge the sample and allow generalizations.

The last independent variable analyzed in the current study was the playing style. The offensive playing style was divided into a counterattack and positional attack, which are constantly mentioned in the literature^48–50^. Significant weak correlations were found between physical parameters and incidence of positional play attacking actions, and no significant correlations were reported in the counterattack plays. In summary, this means that the relations between these variables are non-existent or non-linear. Differences in the effectiveness of attacking styles have been reported in the literature^51^, which could lead to different behavioral patterns (and, hence, differences in physical parameters). However, in the current study, we analyzed the association between the frequency of different attacking styles and the physical parameters. At this point, previous studies showed variations in the physical demands within matches, for example, considering the worst-case scenarios^52,53^. However, the classification of the attacking styles considered the whole match. For this reason, differences in the styles could have been biased by the absence of a play-by-play analysis. Specifically, even if attacking styles could be associated with different physical demands, the analysis of the demands in the whole match makes it hard to detect the associations. Future studies should look at splitting the physical variables considering the classification attacking playing styles by, for example, merging a notational analysis with the GPS data.

The literature has commonly adopted the analysis of the influence of contextual factors on players' physical performance. However, this study has some clear innovative insights. The most relevant strengths are the number of contextual variables simultaneously considered in the current study, the access to a top-level club, and the full-season athletes' follow-up. On the other hand, we acknowledge that the data has been collected from a single club, which requires caution when generalizing the results. Also, due to the high external validity, controlling intervening variables (such as match schedules, congested fixtures, starting eleven, and others) was not possible, which could have biased the results. Future studies in elite soccer are recommended to expand the current insights.

## Conclusions

In summary, matches played against weak opponents presented greater values of SPR distances compared to matches against intermediate and strong opponents. In addition, players covered greater HIR distances when drawing than winning the matches. Matches with assistant coaches presented higher MSEPT compared to coach 1 and coach 2 conditions. Also, players covered greater MSEPT and Acc in matches with coach 2 vs. coach 3. Finally, small positive correlations were observed between positional attack sequences and MSTPT, total distance covered, and Acc. Coaches and practitioners should consider these results when interpreting external load variables during elite Brazilian soccer matches.

## Data Availability

The datasets generated during and analyzed during the current study are available from the corresponding author on reasonable request.

## Abbreviations

W: Weak
I: Intermediatie
S: Strong
L: Loss
D: Draw
W: Win
AU: Arbitraty units
sRPE: Session of rating of perceived exertion
MSTPT: Mean speed relative of total playing time
MSPT: Mean speed relative of effective playing time
MRS: Maximal running speed
TD: Total distance covered
MIR: Moderate intensity running (14.4–19.8 km h^−^^1^)
HIR: High intensity running (19.8–25.2 km h^−^^1^)
SPR: Sprinting (> 25.2 km h^−^^1^)
Acc: High acceleration (> 3 m s^2^)
Dec: High deceleration (< − 3 m s^2^)
